# Gradient moduli lens models: how material properties and application of forces can affect deformation and distributions of stress

**DOI:** 10.1038/srep31171

**Published:** 2016-08-10

**Authors:** Kehao Wang, Demetrios Venetsanos, Jian Wang, Barbara K. Pierscionek

**Affiliations:** 1 Faculty of Science Engineering and Computing, Penrhyn Road, KT1 2EE, Kingston-upon-Thames, UK

## Abstract

The human lens provides one-third of the ocular focussing power and is responsible for altering focus over a range of distances. This ability, termed accommodation, defines the process by which the lens alters shape to increase or decrease ocular refractive power; this is mediated by the ciliary muscle through the zonule. This ability decreases with age such that around the sixth decade of life it is lost rendering the eye unable to focus on near objects. There are two opponent theories that provide an explanation for the mechanism of accommodation; definitive support for either of these requires investigation. This work aims to elucidate how material properties can affect accommodation using Finite Element models based on interferometric measurements of refractive index. Gradients of moduli are created in three models from representative lenses, aged 16, 35 and 48 years. Different forms of zonular attachments are studied to determine which may most closely mimic the physiological form by comparing stress and displacement fields with simulated shape changes to accommodation in living lenses. The results indicate that for models to mimic accommodation in living eyes, the anterior and posterior parts of the zonule need independent force directions. Choice of material properties affects which theory of accommodation is supported.

The eye lens together with the cornea are the optical elements of the eye and refract light to the retina. Whilst the cornea provides two-thirds of the refractive power, the lens is able to adjust its power enabling the eye to focus over a range of distances. This is accomplished by the lens changing shape, a process referred to as accommodation. With age, this accommodating ability gradually diminishes, reducing the near range of focus so that by the sixth decade of life, the eye is no longer able to focus on close objects; this loss of function is termed presbyopia^1^,^2^,^3^. There remains a paucity in the understanding of accommodation and hence of how the relevant components of the eye contribute to the functional loss. Currently there are two opposing theories that purport to explain the accommodative mechanism^1^,^2^,^3^. The significant difference between these theories lies in the mediation of forces in the zonule and the tensions imparted on the different parts of the zonule during accommodation^1^,^2^,^3^. The zonule encircles the equator of the lens and is composed of fibrils that run from the capsule, within which the lens is located, to different parts of the ciliary muscle and are broadly divided into anterior, posterior and equatorial sections^1^,^2^,^3^.

The oldest and more established theory, is that of Helmholtz^1^, who postulated that as the lens changes shape to focus on distant objects, the increased tension in the zonular fibres stretches the lens to a flatter shape to decrease optical power. Conversely, when the lens focusses on objects at close range, decreased tension in zonular fibres allows the lens to assume a more curved shape providing an increase in optical power; the latter is considered a more relaxed form for the lens^1^. Schachar^2^,^3^ hypothesised that the accommodated lens (the form the lens assumes for near vision) requires an increase in tension in the equatorial zonule that causes the lens to assume a spindle shape as the central surfaces become more curved and the peripheral surfaces flatten^2^,^3^. As it is not possible to examine the zonular movements in a living human eye because the iris blocks the view of the equatorial region of the lens, computational modelling, using advanced methods based on measurements of human lenses, offers a means of studying the action of the zonule during accommodation. Comparing the results of such models with those obtained from clinical measurements on live eyes allows an optimisation of model parameters to obtain a physiologically relevant model that will provide a greater understanding of zonular behaviour during accommodation and what may determine the functional loss with age.

The accuracy of any computational model relies on the data used to construct it, in this case on both geometric parameters of the components of the eye involved in accommodation and on the mechanical properties of the lens. The geometrical parameters of curvature and thickness can be obtained from live eyes using clinical biomicrosopic means^4^,^5^,^6^,^7^. Since the lens grows throughout life, with age, its thickness increases and the central anterior and posterior radii of curvature decrease^4^,^5^,^6^,^7^. Material properties have been much harder to ascertain. *In vitro* studies, either with respect to age or across the lens, differ greatly^8^,^9^,^10^,^11^,^12^,^13^,^14^. The seminal work of Fisher provides values of Young’s moduli for both the lens substance and the lens capsule over a wide age range^8^,^15^. Subsequent studies have reported different trends and values^9^,^10^,^11^,^12^,^13^,^14^. Such divergence in measurements may arise from different measurement techniques and inherent assumptions.

There is a varying protein distribution across the lens resulting in the gradient refractive index that contributes to the high degree of image quality^16^. The linear relationship between protein concentration and refractive index is well understood^16^,^17^. The material properties across the lens also vary, as experimental studies^9^,^11^ have shown, but the correlation between these changes and the protein concentration is still unclear. Recently, *in vivo* studies using Brillouin optical microscopy have reported profiles of elasticity along the lens central axis^18^,^19^ that mimic the gradient of refractive index^16^,^17^,^20^, suggesting linear relationships may also exist between mechanical and optical properties.

Finite Element Analysis (FEA) models can be used to test the veracity of measured material properties and a number of FEA models have been suggested^21^,^22^,^23^,^24^,^25^,^26^,^27^,^28^. The present study proposes the most biologically accurate FEA lens models to date, using lens curvatures and material gradients based on experimental data^20^ to test different material properties proposed in previous work^8^,^12^,^15^. The effect of changes in elastic moduli of the lens substance, different levels of stretching forces provided by zonular fibres and various stretching angles of zonular fibres are analysed to determine how they affect the anterior and posterior curvatures of the lens approximated by spheres and conicoids of revolution to determine which theory of accommodation is supported, and how the model results compare to studies on living eyes.

## Results

### Stress patterns

#### Models incorporating Fisher’s data

**Figure 1:**
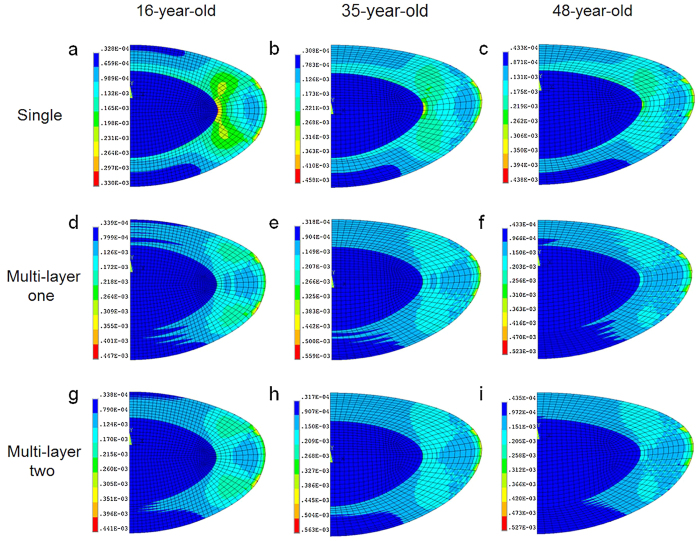
Stress patterns (as von Mises stress in MPa = N/m^2 ^× 10^6^) for human lens models aged 16, 35 and 48 based on optical measurements^20^ and using material properties of Fisher^8^.

Stress patterns for the three different ages of lens models with single values for the cortical modulus are shown in the Fig. 1(a–c). The corresponding stress values of each model are indicated using colour coding (as von Mises stress in MPa) with the colour bar on the left side. The lens nucleus indicated in dark blue has minimal stress values for each lens model. There is a high stress region indicated in green and yellow colours concentrated near the nuclear region of the equator, which is especially marked in the 16-year-old lens. The anterior stress is higher than the posterior stress for all three models.

Figure 1(d–f) shows the three aged models in which the lens cortex has been divided into layers of different moduli based on the gradient index measurement of Bahrami *et al*.^20^. The cortex in the smallest of these lens models, based on the 16-year-old lens^20^, is divided into four layers; the cortical regions in the other two lenses have five layers. Young’s moduli for each model increases by 0.6 MPa increment from the innermost to the outermost layer, maintain a mean value of all layers that is in accordance with the values reported by Fisher^8^; Young’s moduli of the nucleus is constant. Layered stress patterns are observed in the anterior and posterior cortical regions of the 16-year-old lens, and largely in the posterior cortical region for the 35-year-old and the 48-year-old lenses. The stresses at the equatorial cortical region are more evenly distributed than in the single cortex models.

Dividing the cortex further into more layers whilst maintaining the same mean value as reported by Fisher^8^: ten layers for the 16-year-old lens model, twelve layers for the 35-year-old lens model and eleven layers for the 48-year-old lens model, in accordance with the contours of refractive index^20^ gives results shown in Fig. 1(g–i). The stresses are more evenly distributed in all three lens models from this set than in the models with the single value of cortical modulus (Fig. 1a–c) and in those with fewer layers (Fig. 1d–f).

#### Models incorporating Wilde *et al*. ’s data

**Figure 2:**
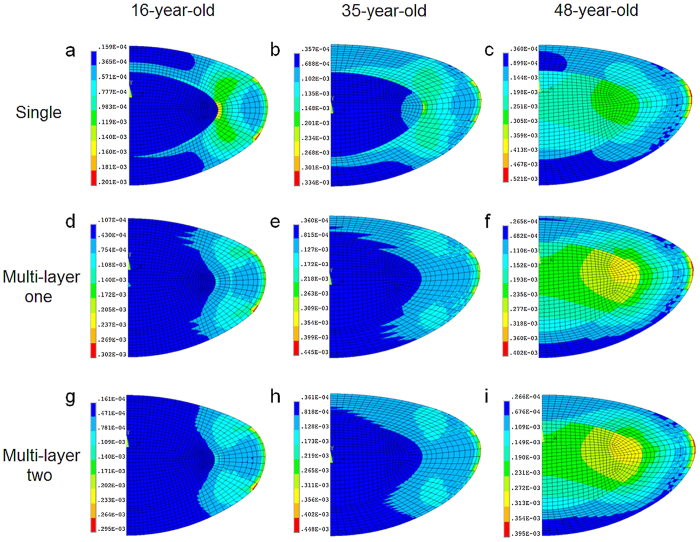
Stress patterns (as von Mises stress in MPa = N/m^2 ^× 10^6^) for human lens models aged 16, 35 and 48 based on optical measurements^20^ and using material properties of Wilde *et al*.^12^.

The stress patterns for lens models incorporating material properties reported by Wilde *et al*.^12^ are displayed in Fig. 2. For single cortex models, the 16-year-old and the 35-year-old lenses display similar stress patterns to those using material properties of Fisher^8^ (Fig. 2a,b). The 48-year-old lens models, however, shows greater differences depending on the moduli used; the model using the elastic moduli of Wilde *et al*.^12^ has a high stress region in the equatorial region of the nucleus with minimal stresses in dark blue colour appearing in the posterior cortex (Fig. 2c).

For models with multiple layers (following the same layered format used to apply the material properties measured by Fisher^8^) the resulting stress patterns are shown in the Fig. 2(d–i). The 16-year-old and 35-year-old lens models do not show the jagged edged stress patterns in the posterior cortex that are seen in the models with the material properties of Fisher^8^ (Fig. 1d–i). The stresses are lower and more diffuse in the models with the material properties of Wilde *et al*.^12^ compared to those of Fisher^8^. Conversely, the two 48-year-old lens models with multiple cortical layers have regions of higher stress particularly in the nuclear equatorial region compared to the respective models using the properties measured by Fisher^8^.

### Lens deformations

The models were subjected to simulated stretching forces of 0.08 N. The sagittal deformations of the nucleus and cortex are listed in Table 1 for the single cortex models as the representative forms since, in terms of deformation, multi-layer models demonstrate similar changes to corresponding single cortical modulus models. Changes in thickness of the nucleus are larger than those of the cortex for all listed models. All three aged lens models using material properties of Fisher^8^ and the two younger lens models using material properties of Wilde *et al*.^12^ have lower elastic moduli in the nucleus than in the cortex. The deformation of the nucleus in these models is almost three to six times larger than that of the cortex. The 48-year-old model using material properties of Wilde *et al*.^12^ has higher elastic moduli in the nucleus than in the cortex; compared to the other age models, this oldest model shows the least deformation.

**Figure 3:**
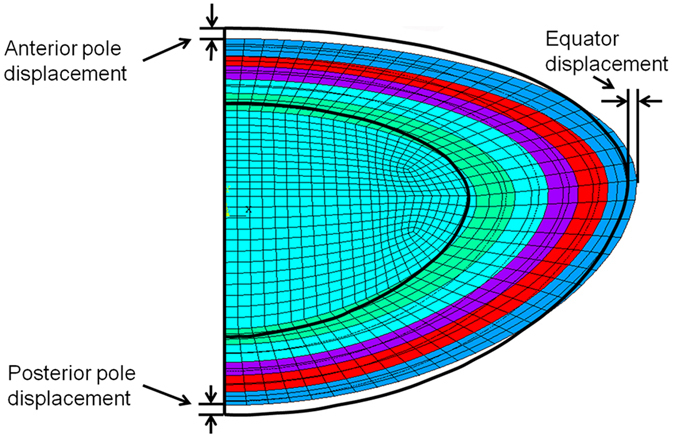
Diagrammatic representation of lens displacements at the equator, anterior and posterior poles.

The displacements of the anterior pole, posterior pole and equator (as illustrated in Fig. 3) for all the lens models are listed in Table 2. With age, the displacements of the poles and the equator decrease. For the two younger lenses, the anterior pole has a greater displacement than the posterior pole for both sets of material properties in single and multi-layer models. There are no significant changes in displacement values when comparing single to multi-layer models (Table 2).

Table 3 gives the central radii of curvature of the lens anterior and posterior surfaces for all the models, including both the anterior and posterior surfaces for the undeformed state and for a deformation caused by a cumulative force of 0.08 N. For the spherical surface approximation, the two younger lens models constructed with the material properties of Fisher^8^ and the youngest lens model with the material properties of Wilde *et al*.^12^ show an increase in anterior central radii of curvature with stretching. For all other models the anterior lens surface becomes steeper as the model is stretched. The posterior surfaces for all the cases become flatter with stretching.

When a conicoid of revolution is used to represent the lens surfaces, only the youngest lens model shows an increase in anterior central radius of curvature with stretching with both sets of material properties. The two older lens models have a decrease in anterior radii of curvature with stretching. The posterior central radii of curvature of all the three aged models using the material properties of Fisher^8^ increase with stretching. Using the material porperties of Wilde *et al*.^12^, the two younger lens models show a decrease and the oldest lens model an increase in posterior central radii of curvature with stretching. Except for the posterior surface of the 48-year-old lens which has a conic constant <−1, all the other surfaces have conic constants larger than 0 (Table 3). There is negligible difference between single and multi-layer models for either set of material properties.

### Modelling of zonular forces

#### Model fitting to anterior curvatures

**Figure 4:**
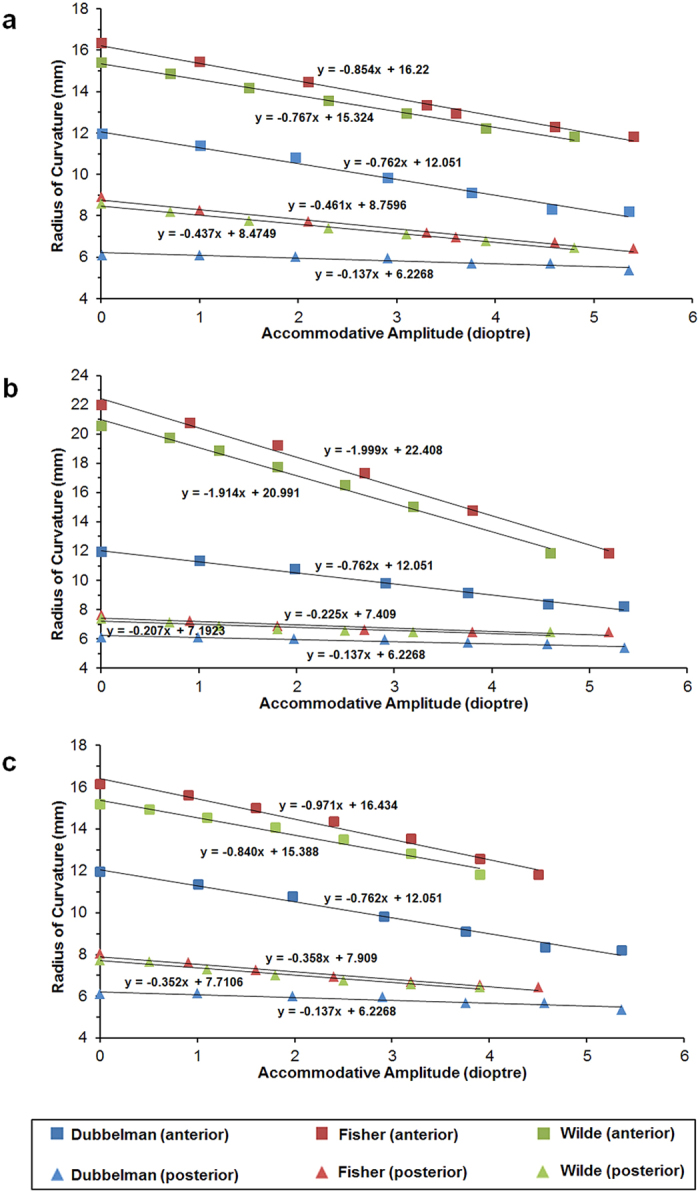
The change in anterior and posterior radii of curvature plotted against accommodative amplitude for the 35-year-old model (**a**) with horizontal zonular stretching (**b**) with zonular fibres shifted anteriorly by 15 degress (**c**) with split zonular fibres.

Applying zonular forces horizontally to the 35-year-old lens model (to enable a closer age-related comparison to clinical measurements from a 29-year-old lens^5^), a linear relationship is found between the change of central optical power and both anterior and posterior radii of curvature (Fig. 4a). The single layer model is used to represent all models as curvature changes and deformations vary little between single and multi-layer models. The slopes are compared with *in vivo* measurements of a 29-year-old lens from the literature^5^ for up to 6D of accommodative amplitude. For the anterior curvature slopes, there are no significant differences between the *in vivo* measurement and the two models with different material properties (p = 0.105 for model using material properties of Fisher^8^ and p = 0.917 for model using material properties of Wilde *et al*.^12^). For the posterior surface, significant differences are found for models with both sets of material properties (p ≪ 0.01).

#### Model fitting to posterior curvatures

A series of models were developed with the zonular fibres altered to different angles to find the model that could best fit the slopes that represent the posterior surface to *in vivo* measurements. The closest fitting slope (Fig. 4b) is provided by the model that has the zonular fibres shifted anteriorly by 15 degrees. For posterior curvature slopes, no significant differences are found between *in vivo* measurements and the two models (p = 0.095 for model using material properties of Fisher^8^ and p = 0.183 for model using material properties of Wilde *et al*.^12^). Notably for this case, the anterior curvature slopes for models using both sets of material properties deviate significantly from the *in vivo* measurements (p ≪ 0.01 for both models).

### Model fitting to both anterior and posterior curvatures

Additional models were developed with the zonular fibres split into three different sections with independent directions of stretch. Figure 4(c) shows the plot of the slopes for the optimal model which has a 20 degree anterior zonular angle, 15 degree equatorial zonular angle and 40 degree posterior zonular angle. For anterior curvature slopes, no significant differences are found between *in vivo* measurements^5^ and either of the models (p = 0.042 for model using material properties of Fisher^8^ and p = 0.651 for model using material property of Wilde *et al*.^12^). Although there are significant differences found for posterior curvature slopes (p ≪ 0.01 for both models), this model provides the balance of closest fits to the *in vivo* measurements for both the anterior and posterior curvatures.

## Discussion

Individual variations in biological tissues and structures can mask ageing effects given variations in genetics, epigenetics and lifestyle factors. This notwithstanding, eye lens growth with age is well defined^29^,^30^,^31^,^32^. Models in this study were developed from data of refractive index obtained using advanced interferometry^20^. This technique enabled the observation of subtle fluctuations in refractive index that had not been shown before^17^,^20^.

Previous FEA models anchored the zonule to a single point^22^,^25^,^26^,^33^,^34^,^35^,^36^,^37^ resulting in non-physiological discontinuities in curvature. Belaidi and Pierscionek^21^ built three-dimensional models and increased the number of zonular fibres to five for each set. In this study, a master-slave nodes mechanism was introduced by applying a number of constraint equations to zonular-capsular attaching points. This mechanism couples the degrees of freedom of the neighbouring nodes so that they follow the movement of each anchorage point, yielding smooth curvatures on deformed lens shapes (Figs 1 and 2). This model has also incorporated independent directions of stretch for the different zonular sections. These characteristics have yielded a model that more closely mimics the physiological condition^38^.

Stach *et al*.^27^ used FEA to model varying complexities of zonular fibres, comparing ten sets with more simplified arrangements such as three zonular fibres and a single stretching point, and concluded that the zonular construction has little impact on lens accommodation^27^. Although Stach *et al*.^27^ suggested that the simplified zonular model is sufficient for numerical modelling, the models developed in the present study have shown that the effect on the optical power can vary significantly depending on the direction of force application (Fig. 4).

Deformations in the nucleus are significantly larger than in the cortical region when the lens shape was changed for all the models with a lower elastic modulus in the nucleus than in the cortex (Table 1). These include all three aged models using the material properties of Fisher^8^ and the two younger lens models using the material properties of Wilde *et al*.^12^. Thickness changes during accommodation have been reported to occur in nuclear region in several clinical studies^4^,^6^,^29^,^39^. Furthermore, as lens thickness decreases with stretching, the movement of the anterior pole is greater than that of the posterior pole, for the 16-year-old lens models and the 35-year-old lens models (Table 2). Similar changes have been reported in *in vivo* studies^29^,^40^. The converse behaviour of the 48-year-old model in terms of anterior and posterior polar movement could be related to the more asymmetric geometry or the anchorage of zonular fibres but it needs to take account of the fact that a lens of this age has very little accommodative capacity left. With a spherical approximation of curvature fitting, lens central anterior radii of curvature increase with stretching for the two younger lens models using the material properties of Fisher^8^ and for the youngest model using the material properties of Wilde *et al*.^12^ (Table 3). This is in accordance with previous clinical studies^5^,^29^,^31^,^40^,^41^,^42^ and supports the theory of Helmholtz^1^. In the case of the two older lens models using the material properties of Wilde *et al*.^12^ and the oldest lens using the material properties of Fisher^8^, anterior central radii of curvature decrease with stretching in support of the theory of Schachar^3^. When a conicoid is used to represent the central surface^43^, the results indicate that for both sets of material properties there is an increase in anterior central radius of curvature with stretching only for the youngest lens model. The conic constant of the posterior surface of the oldest lens model is lower than −1, suggesting that surface is a hyperboloid. The rest surfaces of the lenses are oblate ellipsoid with conic constants larger than 0.

The stress patterns of the single cortex models are similar to the stress patterns found by Belaidi and Pierscionek^21^, showing a high stress region concentrated near the nuclear equatorial pole when the model undergoes simulated stretching.

The material properties used in the models for this study are from experiments that have used centrifugal forces to deform lens samples^8^,^18^,^12^. Other studies have applied sectioning techniques^9^,^11^,^44^ or inserted probes into the lens substance^11^. Samples measured through invasive methods and particularly when lenses have been frozen and thawed^9^,^44^ may yield different mechanical behaviour compared with fresh, intact lenses. Generalised hardening of the lens tissue with age is conceded^9^,^11^,^12^,^13^,^44^,^45^, although there are variations in the measured locations within the lens among those studies.

Variations in material properties of the nucleus and cortex also occur with age^8^,^12^,^13^. Fisher^8^ reported that the elastic modulus of the nucleus is lower than that of the cortex up to 70 years of age at which point Young’s moduli of these two regions become equal. Weeber *et al*.^11^ and Wilde *et al*.^12^ found that the nucleus has lower elastic modulus than the cortex in young lenses and that this reverses after the age of 45 years. Weeber *et al*.^11^ sectioned the lens and inserted the probe at the level of the equatorial plane. Wilde *et al*.^12^ repeated Fisher’s spinning lens test but unlike Fisher^8^ removed the lens capsule before the spinning process^12^. As the capsule helps to maintain the lens shape and hydration, it is possible that some deformation and dehydration may have occurred by its removal. Heys *et al*.^9^ concurred the elastic modulus in the nucleus is lower than the cortex in younger lenses and becomes higher than the cortex in older lenses but reported that the crossover occurs at 30 years of age. Hollman *et al*.^13^ found that Young’s moduli decrease from centre to periphery in middle (about 40 years old) and older (63–70 years old) ages. The study conducted by Bailey *et al*.^14^, uniquely, reported a higher nuclear bulk modulus than that of the cortex, independently of age. The elastic moduli reported by these studies also vary, with some studies reporting Young’s modulus^8^,^13^. others shear modulus^11^,^12^ or bulk modulus^14^.

Applying a stretching force of 0.08 N, as in a previous modelling study^23^, all lens models show decreasing trends in displacements with age (Table 2), confirming an increased resistance of older lenses to stretching forces^46^,^47^ in accordance with the physiological condition of presbyopia. Further analyses of many more lenses across a wider age range are needed to determine whether there is a cross over point where the elastic modulus in the nucleus becomes higher than in the cortex and at what age this may occur.

Weeber *et al*.^48^ divided the lens into 10 concentric regions based on stiffness measurements in the lens equatorial plane^11^ and concluded that the changing stiffness gradient is the cause of presbyopia. Recent studies using Brillouin optical microscopy reported the appearance of a gradient of elasticity in the lens cortex and a relatively flattened region of elasticity in the nuclei of human and porcine lenses^18^,^19^, confirming the assumption made in this work, that the moduli of elasticity can be modelled adhering to the form of the refractive index gradient.

From the results in our study, as the number of cortical layers increases, there are no significant variations in either deformation changes nor in central curvature between the models with different cortical layers (Tables 2 and 3). The stiffness variations within the lens only have an effect on internal stress distributions. The more evenly distributed stresses in the cortical regions of multi-layer models, compared with single cortex models, demonstrate that the material property within the cortex is unlikely to be a constant value, as high stress concentrations in biological tissues could be detrimental physiologically.

Anatomical studies have shown that there are three sets of zonular fibres that are attached to the lens capsule anteriorly, equatorially and posteriorly^49^,^50^. Most of the *in vivo* studies on lens accommodation focused on the changes in the central region^5^,^6^,^7^,^29^,^51^,^52^,^53^,^54^. The linear slopes for both the models with a single stretching point and the models with three stretching points vary slightly from clinical measurements. This small difference may arise from a number of sources. The geometric data adopted for the present models are from measurements on post-mortem lenses so that the whole lens is in a stress-free state. The *in vivo* lens is under a small amount of tension even at fully accommodated states. Factors like gravity, pressure and buoyancy from the vitreous and aqueous humours are excluded in post-mortem lenses measurements. Individual variations on lens geometry and insertion regions of zonular fibres in lens peripheral region can also influence the curvature changes during accommodation.

## Conclusion

The use of human lens models subjected to simulated stretching indicates that stress distributions and deformations depend on material properties and on the surface shapes chosen. The choice of material and shape parameters can alter which theory of accommodation is supported. Models that allow for various zonular sections to be stretched in different directions more closely mimic the physiological condition and accommodative amplitudes derived from such modelling can be compared to clinical findings. Further analysis needs to be performed on more lenses with different distributions of material properties and with different degrees and directions of stretching force in order to optimise models and to refine the choice of parameters so that they can better represent the real lens.

## Methods

### Model components

A set of 20 three-dimensional quarter models were developed using ANSYS mechanical APDL version 16.0. These models were based on measurements^15^ of three human lenses aged 16, 35 and 48 years old. Each model consisted of seven different parts: lens nucleus, lens cortex, capsule, anterior zonular fibres, equatorial zonular fibres and posterior zonular fibres. Capsular thicknesses, for the 16, 35 and 48 aged lenses were 13 μm, 15 μm and 17 μm respectively as reported in the literature for lenses of these ages^15^.

### Material properties

The material for each part was assumed to be linear elastic, isotropic and homogenous. The elastic moduli assigned to the nucleus and cortex (Table 1) were taken from measurements of Fisher^8^ and Wilde *et al*.^12^. Poisson’s ratio of 0.49 was used for the lens cortex and nucleus^37^,^48^. Young’s moduli were 5.87 MPa, 4.90 MPa and 4.20 MPa for the capsule of the 16, 35 and 48 aged lenses^15^ respectively, and 0.35 MPa for the zonular fibres^55^. Poisson’s ratio for both the capsule and zonular fibres was 0.47^15^,^56^.

### Finite Element Model Development

The lens nucleus and cortex were considered as solid bodies and were meshed using 20-node brick elements (ANSYS element type: SOLID 186). Mixed u-p element formulation method was employed to avoid locking phenomenon for nearly incompressible material behaviour. The capsule was treated as a membrane and was meshed using 8-node shell elements (ANSYS element type: SHELL 281). The zonular fibres were modelled as 2-node 3D spar elements, with three translational degrees of freedom at each node and with the ability to carry uniaxial tension only (ANSYS element type: LINK 180). The total number of elements for the examined models ranged from 16923 to 23523, while the total number of nodes ranged from 100632 to 139718. Non-linear geometrical analyses were performed for all models.

### Analysis of stress patterns

**Figure 5:**
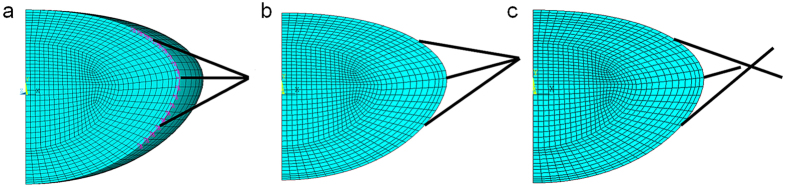
Details of the model showing (**a**) coupling of zonular anchorage points on the capsule to neighbouring nodes (**b**) zonular fibres bound to a single stretching point shifted anteriorly (**c**) zonular fibres with different stretching directions.

An accumulative force of 0.08 N was applied to all lens models, which was a compromise between the ranges of 0.08 N and 0.1 N as reported by Burd *et al*.^22^ and the stretching force of 0.06 N estimated by Hermans *et al*.^24^. The 0.08 N force was evenly distributed to 160 sets of zonular fibres that encircled the lens equator, each set comprising one anterior, one equatorial and one posterior zonule. The length of the zonular fibres were taken from Strenk *et al*.^57^, namely 2.5 mm, 2.0 mm and 1.8 mm for the lenses aged 16, 35 and 48 years old, respectively. In order to model the complex anchorage of the zonules on the capsule, a number of constraint equations were introduced so that the degrees of freedom of the point of anchorage were properly coupled to the degrees of freedom of neighbouring nodes. This coupling is shown in Fig. 5(a), where the three anchorage points of the zonular fibres on the capsule were coupled with neighbouring nodes marked in purple.

Models with a single cortical layer were examined using the material properties reported by Fisher^8^ and Wilde *et al*.^12^. Models with multiple cortical layers with gradient elastic moduli were created based on the contours of refractive index of each particular lens^20^. The moduli in the cortex of these multi-layer models increased from the innermost to the outermost layers, maintain a mean value of all the cortical layers in accordance with previous measurements^8^,^12^.

### Fitting to clinical measurements

Six levels of accummulative force: 0.080 N, 0.128 N, 0.160 N, 0.240 N, 0.320 N and 0.400 N, were applied to the 35-year-old lens model as shown in Fig. 5(a). The sagittal thicknesses of the lens, together with radii of curvature of the lens anterior and posterior surfaces were extracted from both the undeformed and deformed states within the lens central 3mm diameter zone. For radii of curvature calculated with a spherical surface approximation circle fitting method^58^ using MatLab version 2013b was used. Central Optical Powers (COP) of the lens models were calculated based on equation (1)^22^,^36^:


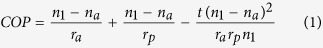


where 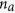 = 1.336, is the refractive index of aqueous humour, 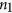 = 1.42 is the estimated overall refractive index of the lens which is a representative equivalent refractive index, 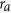 and 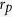 are the anterior and posterior radii of curvature, t is the sagittal thickness of the lens.

To represent the lens surface with a conicoid of revolution, the radius of curvature and conic constant were fitted using MatLab version 2013b based on equation (2) obtained from a ray tracing study^43^:


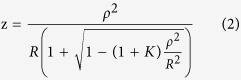


where R and K are the radius of curvature and conic constant of the surface respectively and 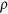 is the distance from the optical (z) axis.

The relationship between both the anterior and posterior radii of curvatures and the change in COP (or accommodative amplitude) were compared with *in vivo* measurements from a lens aged 29 years old^5^. The slopes of the linear regression line that depicts the relationship between the radii of curvature and accommodative amplitude were determined in Excel 2010 and were compared using standard t-test for two independent samples. Different zonular stretching angles were then generated and applied in combination with different levels of stretching forces (as represented in Fig. 5b) and subsequently tested in different directions for angles of: 5, 10 and 15 degrees (anteriorly); 10 and 20 degrees (posteriorly).

Subsequent models were developed by providing the anterior, equatorial and posterior zonular fibres with abilities to move in different directions (as represented in Fig. 5c). A series of combinations were simulated with 5 degree increments in the angles of each of the three zonular fibres at a time to find the optimal model that would most closely fit with clinical measurements^5^.

## Additional Information

**How to cite this article**: Wang, K. *et al*. Gradient moduli lens models: how material properties and application of forces can affect deformation and distributions of stress. *Sci. Rep*. **6**, 31171; doi: 10.1038/srep31171 (2016).

## Figures and Tables

**Table 1:** Sagittal deformations and material properties of the nucleus and cortex.

		16 years old		35 years old		48 years old	
		nucleus	cortex	nucleus	cortex	nucleus	cortex
Fisher^8^	Deformation (mm)	0.2761	0.0586	0.1829	0.0493	0.1367	0.0447
	Young’s modulus (kPa)	0.50	2.40	0.60	3.70	1.10	4.00
Wilde *et al*.^12^	Deformation (mm)	0.3770	0.0638	0.1694	0.0524	0.0653	0.0358
	Shear modulus (kPa)	0.06	0.32	0.26	0.89	2.67	1.22

**Table 2:** Displacements of lens equaotor, anterior pole and posterior pole.

		Fisher^8^			Wilde *et al*.^12^		
Unit/mm		Single	Multi-layer one	Multi-layer two	Single	Multi-layer one	Multi-layer two
16 years old	Equator	0.1187	0.1193	0.1191	0.1408	0.1393	0.1388
	Anterior	0.1901	0.1914	0.1909	0.2540	0.2518	0.2504
	Posterior	0.1446	0.1455	0.1452	0.1868	0.1849	0.1840
35 years old	Equator	0.0961	0.0964	0.0963	0.0984	0.0991	0.0988
	Anterior	0.1187	0.1199	0.1197	0.1128	0.1149	0.1143
	Posterior	0.1135	0.1139	0.1139	0.1090	0.1096	0.1094
48 years old	Equator	0.0903	0.0906	0.0903	0.0861	0.0860	0.0862
	Anterior	0.0811	0.0817	0.0817	0.0347	0.0344	0.0344
	Posterior	0.1004	0.1005	0.1005	0.0664	0.0668	0.0667

**Table 3:** Central radius of curvature (R) and conic constant (K) of both anterior and posterior surfaces.

			Un-deformed model	Fisher^8^			Wilde *et al*.^12^		
				Single	Multi-layer one	Multi-layer two	Single	Multi-layer one	Multi-layer two
Spherical approximation									
16 year old	Anterior	R	7.15	7.88	7.85	7.86	7.93	7.87	7.88
	Posterior	R	5.91	6.14	6.11	6.12	6.05	6.02	6.03
35 year old	Anterior	R	11.81	12.29	12.21	12.23	11.78	11.68	11.71
	Posterior	R	6.40	6.70	6.71	6.72	6.63	6.59	6.60
48 year old	Anterior	R	13.82	13.58	13.50	13.54	12.73	12.83	12.80
	Posterior	R	6.68	6.91	6.90	6.91	6.71	6.73	6.73
Conicoid approximation									
16 year old	Anterior	R	7.42	7.90	7.86	7.88	7.87	7.82	7.83
		K	1.99	0.18	0.15	0.16	0.43	0.43	0.39
	Posterior	R	6.23	6.26	6.23	6.24	6.15	6.13	6.13
		K	2.01	0.75	0.74	0.76	0.61	0.67	0.66
35 year old	Anterior	R	13.81	12.94	12.86	12.88	12.33	12.19	12.22
		K	25.36	7.91	7.75	7.90	6.28	5.86	5.95
	Posterior	R	6.99	7.02	6.99	6.99	6.88	6.83	6.84
		K	3.91	2.09	2.04	2.04	1.81	1.72	1.74
48 year old	Anterior	R	15.94	14.19	14.10	14.14	13.79	13.91	13.87
		K	32.45	9.00	8.67	8.49	14.82	15.20	15.10
	Posterior	R	6.29	6.66	6.64	6.65	6.52	6.54	6.54
		K	−2.40	−1.67	−1.64	−1.67	−1.18	−1.22	−1.19
